# Scope of professional roles for genetic counsellors and clinical geneticists in the United Kingdom: Position on behalf of the Association of Genetic Nurses and Counsellors and the Clinical Genetics Society

**DOI:** 10.1038/s41431-022-01214-7

**Published:** 2022-11-01

**Authors:** Anna Middleton, Nicola Taverner, Catherine Houghton, Sarah Smithson, Meena Balasubramanian, Frances Elmslie

**Affiliations:** 1 Engagement and Society, Wellcome Connecting Science, Wellcome Genome Campus, Hinxton, Cambridge, UK; 2 Kavli Centre for Ethics, Science, and the Public, Faculty of Education, University of Cambridge, Cambridge, UK; 3 School of Medicine, Cardiff University and the All Wales Medical Genomics Service, Cardiff, UK; 4 Liverpool Centre for Genomic Medicine, Liverpool Women’s NHS Foundation Trust, Liverpool, UK; 5 Department of Clinical Genetics, University Hospitals Bristol and Weston NHS Foundation Trust, Bristol, UK; 6 Faculty of Health Sciences, University of Bristol, Bristol, UK; 7 Department of Oncology and Metabolism, University of Sheffield, Sheffield, UK; 8 Sheffield Clinical Genetics Service, Sheffield Children’s NHS Foundation Trust, Sheffield, UK; 9 South West Thames Centre for Genomics, St George’s University Hospitals NHS Foundation Trust, London, UK

**Keywords:** Health care, Genetics

## Abstract

This document is written on behalf of the two professional bodies in the United Kingdom that represent genetic counsellors (the Association of Genetic Nurses and Counsellors) and clinical geneticists (the Clinical Genetics Society) and aims to support multidisciplinary working of these professional groups highlighting within a quick-reference format, areas of shared practice and the distinctions between role profiles for a Consultant Clinical Geneticist, Principal/Consultant Genetic Counsellor and the new support role that we have termed ‘Genomic Associate’, see AGNC career structure [1]. This builds on published documents that articulate the scope of practice of the clinical genetics workforce [2] and specifically the genetic counsellor [3] and clinical geneticist [4] roles.

In the United Kingdom clinical geneticists are medically qualified Members/Fellows of the Royal College Physicians or equivalent, where Clinical Genetics is an affiliated medical specialty. Genomic or genetic counsellors are allied health professionals with Masters level accreditation from the Genetic Counsellor Registration Board included in the Academy for Healthcare Science register and clinical scientists (genomic counselling specialty) accredited by the Health and Care Professions Council.

We acknowledge there is currently variability in these roles between NHS trusts and exceptions where the scope of practice for one professional group exceeds what is provided below in Fig. 1.

**Fig. 1:**
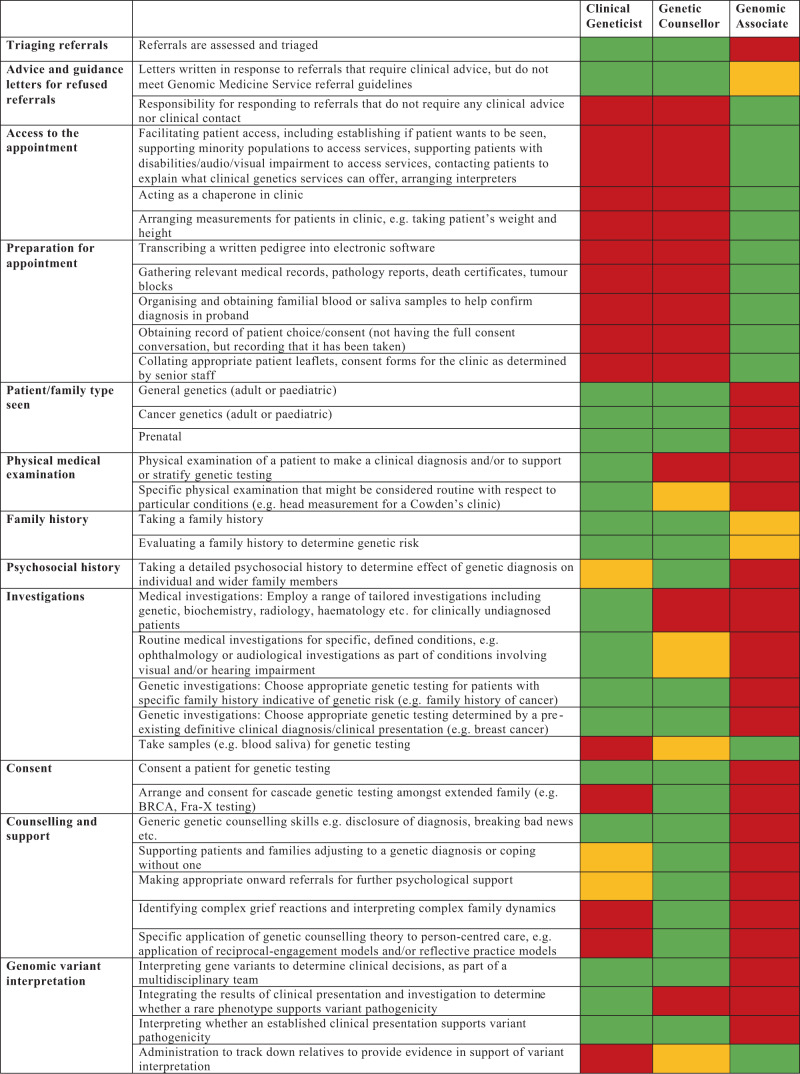

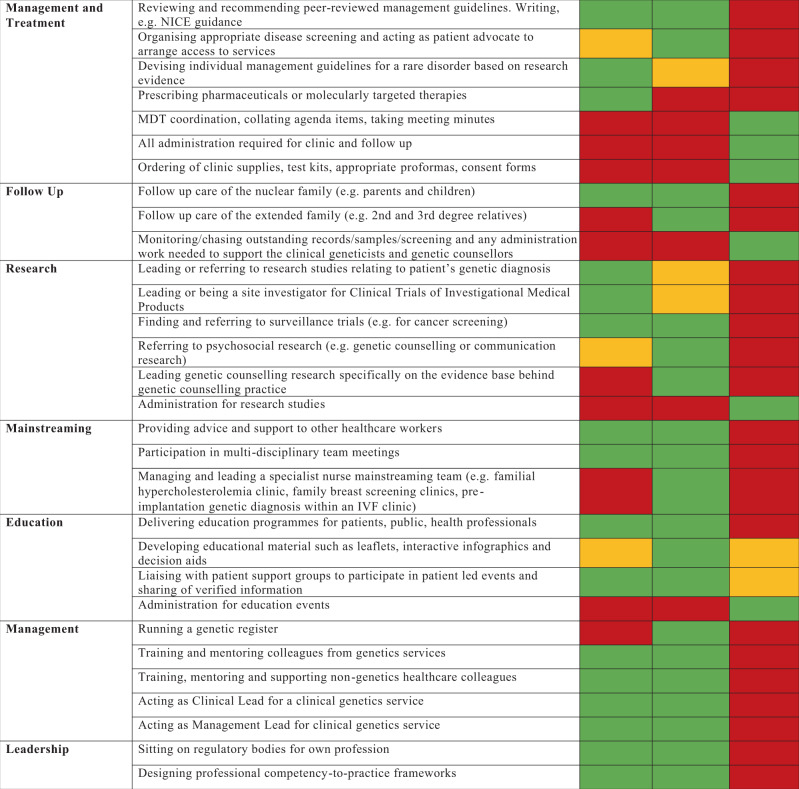
Scope of professional roles for clinical geneticist, genetic counsellor and genomic associate in the United Kingdom. The colour coding provides a guide to the professional group providing each aspect of service: green = routinely within the scope of practice, amber = within the scope of practice for some professionals, but not for the majority, red = outside of the scope of routine practice.

In Fig. 1 the roles are deliberately forward looking, i.e. they acknowledge that there are some areas of practice that may have traditionally been performed by one professional group, can now be shared with or devolved to other groups. Broadly speaking, the clinical geneticist leads on diagnostics and therapeutics and the genetic counsellor leads on psychosocial issues and care of the extended family. Both groups have skills and training in clinical genetics and there is much cross over between roles. The genomic associate leads on administrative support for the clinic, the patient and the clinical activities of the clinical geneticist and genetic counsellor. The genomic associate is part of the genetic counsellor career structure and has a clinical role that is different to a secretary; it is a position that has already been discussed in relation to the Genomics Service Specification for the National Health Service in the United Kingdom.
